# Beyond buzzwords: fostering interdisciplinary and collaborative global health research in Germany and beyond

**DOI:** 10.1080/16549716.2024.2408884

**Published:** 2024-10-21

**Authors:** Maeve Cook-Deegan, Kerem Böge, Walter Bruchhausen, Mizeck Chagunda, Medha Chaturvedi, Meral Esen, Johanna Hanefeld, Beate Kampmann, Carsten Köhler, Charlotte Köhler, Francis Osei, Clarissa Prazeres da Costa, Eva Rehfuess, Thirumalaisamy P. Velavan, Nora Anton, on behalf of the GLOHRA Steering Committee*

**Affiliations:** a Charité Center for Global Health, Charité Universitätsmedizin Berlin, Berlin, Germany; b Department for Psychiatry and Psychotherapy, Charité Universitätsmedizin Berlin, Berlin, Germany; c Section Global Health, University of Bonn, Bonn, Germany; d Department of Animal Breeding and Husbandry in the Tropics and Subtropics, University of Hohenheim, Hohenheim, Germany; e Department of Geography, Heidelberg University, Heidelberg, Germany; f Institute of Tropical Medicine, Travel Medicine & Human Parasitology, Tübingen University Hospital, Tübingen, Germany; g Robert Koch Institute, Centre for International Health Protection (ZIG), Berlin, Germany; h Institute for Medical Microbiology, Immunology and Hygiene, Technical University of Munich, Munich, Germany; i Faculty of Business Administration, European University Viadrina Frankfurt (Oder), Frankfurt (Oder), Germany; j Department for Public Health and Health Services Research, Ludwig-Maximilians-Universität München, Munich, Germany; k Professorship of Medical Sociology and Psychobiology, University of Potsdam, Potsdam, Germany

**Keywords:** Interdisciplinary research, cross-sector research, capacity building, global health research, research network

## Abstract

**Background:**

Germany has increased its political and financial commitment for global health, but this needs to be backed by a robust global health research ecosystem with strong partnerships in low- and middle-income countries (LMICs).

**Objective:**

This article suggests pathways for empowering researchers to operate beyond their disciplinary silos and strengthen partnerships across sectors and countries. The authors identify barriers and enablers of operations from a nascent research network in Germany, trusting that this experience can inform other initiatives seeking to stoke interdisciplinary and collaborative global health research.

**Methods:**

This article represents the culmination of extensive reflections spanning the initial four years of the German Alliance for Global Health Research (GLOHRA). The insights have additionally been informed by an analysis of publicly available reports, internal procedural records, and externally conducted studies based on interviews with researchers and policymakers.

**Results:**

GLOHRA has developed a toolbox of practices that foster interdisciplinary research and support capacity-building. Insights indicate that highly interdisciplinary and diverse governance structures and seed-funding for interdisciplinary and cross-sector research with appropriate review processes represent a critical step for achieving these aims. Additionally, inclusive training sessions and networking events help to bridge disciplinary boundaries, equipping researchers to envision the broader context of their work.

**Conclusions:**

Despite achievements, challenges persist. Wider support, especially from universities and research institutions, is necessary to make global health research an attractive career path and to reduce bureaucratic barriers for collaborators in LMICs. Sustained, longer-term federal funding mechanisms will also be essential for ongoing progress.

## Paper context

- **Main findings:** Germany’s increased commitment to global health includes federal support for initiatives such as the nascent German Alliance for Global Health Research, a 1000+ member network which aims to foster interdisciplinary and collaborative global health research.
- **Added knowledge:** This article reveals the structures, funding mechanisms, and capacity-building initiatives that have bolstered progress on network aims.
- **Global health impact for policy and action:** Challenges persist and there is a need for sustained federal funding and wider institutional support for global health careers as well as improved conditions to support more equitable research endeavors with partners in low- and middle-income countries.

## Background

Germany has increased its political and financial commitments to global health, partially in response to criticisms of its fragmented global health policies [1,2], insufficient support of the global health academic workforce [3], its relative lag in global health research and development (R&D) [4], and limited interdisciplinary exchange [5]. In 2020–2021 Germany was the largest donor to the World Health Organization (WHO), contributing approximately 1.26 billion USD [6]. The World Health Summit has been running for over ten years and has helped generate a broader interest in global health in Germany, as evidenced by the opening of offices in Berlin by the Bill & Melinda Gates Foundation, Wellcome Trust and WHO Hub for Pandemic and Epidemic Intelligence. Although there is clear political will to advance work on global health, significant backing is needed to develop a thriving ecosystem for R&D that includes strong partnerships in low- and middle-income countries (LMICs), and to improve academic and financial support for early career researchers seeking to pursue a career in global health.

In 2020, building on an earlier concept for shaping global health, the first German Federal Global Health Strategy [7] was published to outline the country’s political commitments to global health and identify strategic priorities. The strategy underscores the importance of R&D, including support for the establishment of the German Alliance for Global Health Research (GLOHRA). GLOHRA is a networking platform to support interdisciplinary, cross-sector, and international research in global health. Since its inception in February 2020, GLOHRA has grown to more than 1000 members [see Annex A] – researchers of more than 75 nationalities working in public research institutions across Germany.

## Objective

### Mechanisms that support interdisciplinarity and strengthen partnerships across sectors and countries: lessons from a global health research network in Germany

GLOHRA was born out of a shared vision to support collaboration in global health research. In February 2019, around 50 researchers from universities and research institutions across Germany convened as a working group to review the role of academia in global health research and policy. The working group responded to a call for proposals from the Federal Ministry of Education and Research (BMBF) and successfully secured funding for what is now GLOHRA, coordinated by a secretariat at Charité - Universitaetsmedizin Berlin.

In the process of shaping the procedures and missions of GLOHRA, we often looked to other networks for inspiration such as the Consortium of Universities for Global Health (CUGH), based in the United States, or to leading global health academic institutions such as the London School of Hygiene and Tropical Medicine (LSHTM), based in the United Kingdom. While these initial insights were valuable, we understood quickly that our focus on interdisciplinarity and the nuances of our ecosystem, including public funding structures, differ from other networks. GLOHRA operations were adapted to our own aims and context. With a shared recognition for the complexity of global health challenges, we sought to find ways to address this by including a broad spectrum of perspectives to enrich approaches to global health research. Based on early experiences of this initiative, we seek to offer key reflections on implementing and managing measures that encourage truly interdisciplinary^1^^1^While we note that interdisciplinary research can be defined in many different ways, we will note here how we have operationalized the term in the FAQs on our website: “For our collaborative research funding lines, i.e. interdisciplinary and cross-sector projects, we expect that project applicants represent at least two of GLOHRA’s research areas, i.e. biomedical science, public health research, social sciences, and humanities, as well as engineering and other sciences. […].We hope that applicants will not interpret this as a minimum technical requirement to meet, but rather as an invitation to integrate approaches from multiple specialties and perspectives throughout the design and execution phases of their research project. We love to see medical doctors working with engineers, statisticians with social anthropologists, and nutrition scientists with health systems researchers. For example, projects could scrutinize a particular problem from multiple perspectives using a mix of methods. They could also involve complementary disciplines and partners early on in the process. This will improve the chances that other researchers, policymakers, communities, or companies will take up the knowledge, technology or intervention generated by the project.” and collaborative research, which may be useful to other networks with similar aims.

## Methods

This article is a joint contribution of the GLOHRA secretariat and steering committee, building on extensive, iterative reflections over the last four years. It also draws on internal documents (e.g. minutes from strategic meetings, project evaluation forms) and publicly available reports (e.g. policy statements [8]). This includes the GLOHRA Engaged Study [9], a report on maximizing the impact of Germany’s role in global health, involving 35 expert interviews with key research and policy stakeholders as well as a survey on the enablers and barriers of practicing global health research in Germany, completed by 103 members. This article represents the culmination of our key insights.

## Results

### Setting up an interdisciplinary network requires governance structures that ensure diverse perspectives

**Figure 1.**
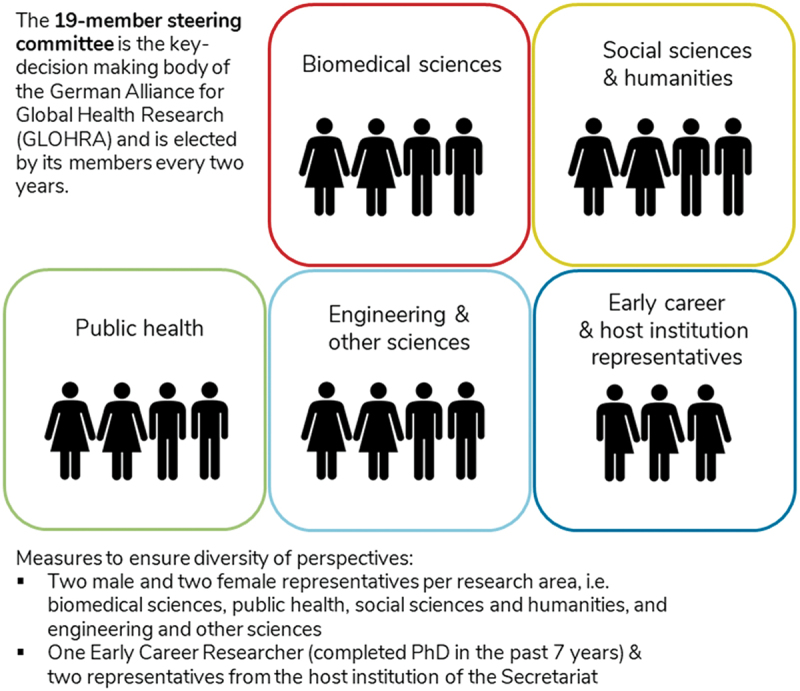
Governance structures of the GLOHRA steering committee.

In the formative stages of GLOHRA, the founding members emphasized the importance of integrating diverse disciplines – there was no interest in duplicating existing initiatives which focused on public health, tropical medicine, or medical anthropology. Accordingly, quotas were established for the steering committee: there would be two female and two male representatives from each of the four designated research areas, i.e., biomedical sciences, public health, social sciences and humanities, and engineering and other sciences [see Figure 1]. The steering committee is elected by members every two years and serves voluntarily without remuneration as the strategic decision-making body as well as the grant selection committee of GLOHRA. This early decision to ensure interdisciplinarity at a structural level had a considerable influence on all subsequent decisions and processes. The steering committee has included members who are originally from Germany, India, Colombia, Ghana, Malawi, Nepal, and Vietnam. All career levels, from doctoral students to full professors, are represented. Together with the secretariat, these members collectively form the leadership team.

This embrace of interdisciplinary governance has brought together different communities, extending beyond the ‘usual suspects’ from the biomedical sciences to include agricultural scientists, climate researchers, engineers, urban planners, and political scientists. The regular opportunities for exchange have built trust across disciplines and institutions. They have furthermore facilitated the development of a joint set of values of what global health research should look like (e.g. in terms of equity, transparency, impact, partnerships, and ecological sustainability), including concrete suggestions on how the overall framework conditions for global health research in Germany could be improved [8,9].

### Promoting interdisciplinary and cross-sector research collaboration with incentives and suitable review processes

GLOHRA has established a toolbox of practices to incentivize research collaboration across disciplines and sectors, including seed funding for research projects as well as support for networking and scientific workshops. Requirements for research funding were set up to guarantee that GLOHRA-supported research projects would be collaborative in nature. In the global health field, research funding that is earmarked for a particular disease is commonplace. Yet, we have made a deliberate decision to embrace a more bottom-up approach where the topic is chosen freely by applicants and instead stipulate different types of consortia, notably **interdisciplinary pilot projects** and **cross-sector funding**.^2^^2^In the GLOHRA **interdisciplinary pilot project** funding line, applicants may only apply if their team includes researchers from at least two different institutions in Germany and represents at least two research areas, for example, a microbiologist and computer scientist. The **cross-sector funding** line further requires the inclusion of a non-academic partner. The minimum consortium stipulation sends an impulse to applicants to establish new mechanisms of cooperation. Indeed, funding which supports research competencies and allows for adaptive and innovative partnerships may have more long-term impact compared to traditional disease-specific funding models [10].

**Figure 2.**
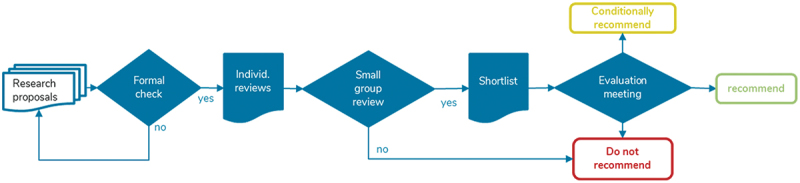
GLOHRA interdisciplinary review process. After submitting individual evaluation reports, small groups representing at least three of the four research areas meet to discuss their impressions and decide whether to shortlist projects or not. The entire steering committee then makes a final decision regarding which of the short-listed projects to recommend.

Establishing an effective, rigorous, and not-too-laborious interdisciplinary review process has turned out to be the most challenging task. This process has been refined into a multi-staged approach, beginning with a written evaluation, followed by small evaluator group discussions and full steering committee discussions [11] [see Figure 2]. The review process ensures that projects are assessed in an integrated way instead of reviewers focusing ‘mainly on their own respective field and thus selectively notic[ing] deficiencies of interdisciplinary proposals rather than their strengths’ [12]. Despite hailing from different academic backgrounds, reviewers have consistently managed to reach consensus-based decisions on recommended projects. This suggests that a shared recognition of high-quality proposals can be reached based on shared values and priorities.

This overall constellation has allowed investigators to test creative combinations of topics and methods that holistically address global health challenges, building bridges between communities.^3^^3^One interdisciplinary pilot project [see RESAMP in Annex B], for example, involves experts from the environmental sciences, public health, and infectious disease researchers. The team is testing the feasibility of introducing an aquatic species into rice fields in Madagascar to reduce the transmission of schistosomiasis, a parasitic disease which is highly prevalent in the country. So far, more than 25 interdisciplinary research projects have been supported by GLOHRA, involving about 70 institutions across 20 countries including Germany, of which 18 are LMIC countries [see Annex B]. Additional funding for **networking** formats as well as **scientific workshops** allows project leaders and members of the GLOHRA community, including early career researchers, to share best practices, experiences and to cross-pollinate.

The results of an external evaluation [13] confirm that GLOHRA has helped to improve the German research system for global health research in recent years. GLOHRA bundles the commitment of numerous German universities and research institutes and contributes to the institutionalization of global health research in Germany. In terms of the international visibility of German global health research, GLOHRA has mainly had an indirect effect so far, i.e., via research projects.

### Recognizing the need to support capacity building in global health research

Early on, GLOHRA leadership emphasized the importance of capacity-building measures for supporting global health research. In Germany, political responsibility for the education system – including higher education – is almost exclusively a matter for the Länder (highly autonomous states; Germany has 16 Länder). As a national research-oriented network, GLOHRA’s mandate is therefore focused on building capacity among early career researchers, mainly doctoral and post-doctoral students. Studies have shown that global health education at medical universities in Germany is not commonplace [14] and structured doctoral training programs for global health research in Germany can be counted on one hand. Recognizing this relative scarcity of options as well as the inherent need for interdisciplinary approaches in global health topics, the ‘Global Health Academy’, a GLOHRA initiative, was established to support early-career researchers, with around four trainings each year as well as a monthly interdisciplinary digital lecture series which is regularly attended by about 50 participants. These activities are designed by and for the community and have attracted an international audience, with many participants and speakers from LMICs. A project funding line for **global health postdoc fellowships** is also available to support career advancement. The implementation of these and other measures aims to fill gaps in the current academic landscape [15] to support early-career researchers. This has led to higher engagement, to development opportunities and has provided a general space for exchange which did not previously exist.

Beyond bolstering the academic landscape, there is a growing advocacy for embedding capacity-building measures in global health research to support local ownership and research culture, enable adaptations based on local contexts, and result in more sustainable solutions [16–18]. This is also expected in GLOHRA projects and the call for research projects stipulates that ‘GLOHRA especially intends to support projects that link scientific excellence with the domains of capacity building, science-based policy advice, teaching, implementation and/or clinical practice [11].’ In 2022–2023, the Federal Ministry for Economic Cooperation and Development (BMZ) funded projects for partnerships and training in health as well as additional ‘booster’-funds for already running research projects to support measures which translated findings into practice and policy. With about EUR 700,000, eight projects in eight countries hosted 61 events involving more than 3400 participants, 98% of whom were from LMICs [19]. This support for capacity-building measures has been explicitly praised by GLOHRA project partners in LMICs, and Dr. Robert Kalyesubula, a project lead in Uganda reflected ‘we keep on coming up against challenges and then together we solve them. In the process, we’ve been able to do advanced research in a rural area and build local teams who know how to do social science research and how to apply for and manage grants without international support’ [20]. Capacity-building measures help to ensure that hard and soft skills (e.g. data collection, grant writing) carry on beyond the scope of the research project and reach beyond those directly involved with undertaking the research.

## Conclusions

### Lessons learned

The growth of GLOHRA as a research network has unveiled both its potential and operational challenges. As mentioned, the review process for research projects has been particularly labor-intensive, especially because the number of applications has more than tripled over the seven calls for proposals, from 14 to 51 [see Annex C]. While the research projects have demanded substantial resources, membership growth has developed organically and has surpassed initial expectations [see Annex A]. Furthermore, the member-driven trainings and workshops have resulted in a vibrant variety of topics and methods, and the relatively modest sums of up to 20,000 EUR have acted as a launching pad for early-career researchers to organize events or to initiate discussions on research topics that could benefit from interdisciplinary exchange.

One point of contention relates to funding international project partners since current stipulations do not allow direct funding of research partners or organizations outside of Germany. This means that international project partners are administratively viewed as sub-contractors, with their ‘services’ subject to Value Added Tax (VAT), which is a difficult starting point for ‘equitable partnerships’ and has required significant effort to clarify and manage.

We have sought to balance advancing our aims with ensuring realistic contributions from the steering committee, who are volunteering their time, and the resource-limited secretariat. Through ongoing procedural adjustments, we have made significant progress toward achieving this equilibrium. However, it is important to note that certain processes, like the application review process, likely require further refinement to better accommodate the unexpected growth in the number of research proposals we receive.

### Path ahead

Germany’s increasing financial and political commitments indicate a desire to support global health activities, including research and innovation. GLOHRA has enjoyed a warm reception from the research community; membership is steadily rising with an even greater increase in applications for research projects and capacity-building offers. Further, GLOHRA has received support from two federal ministries; initially supported with funds by the BMBF only, the BMZ was also motivated to support additional GLOHRA measures to strengthen cooperation with partners in LMICs. This represents an important step towards better coordination and reducing fragmentation in global health activities. A Memorandum of Understanding with the Global Health Hub Germany was also formalized in 2023 in an effort to better link science and policy action in global health.

GLOHRA is committed to forging new mechanisms of cooperation across institutions, disciplines, sectors, and countries. Yet, sustaining and expanding on this work will require broader advocacy and financial support from government initiatives and universities [21], including efforts to increase the attractiveness of a global health career path. Equally, important is the need to push for equitable partnerships with collaborators in LMICs. To this end, the GLOHRA steering committee regularly engages with policymakers and advocates for specific measures to improve conditions for partnerships with LMICs [8,22]. Our collective responsibility lies in advocating for stronger coordination, fostering an ecosystem where innovative ideas thrive and translate into real-world impact. We understand that context is critical when it comes to developing scientific communities and different conditions will demand nuanced approaches. Nevertheless, we are hopeful that GLOHRA’s experience and the mechanisms developed will be useful for existing and emerging organizations and networks that seek to stoke interdisciplinary and collaborative research.

## Annex

### AnnexA. GLOHRA Membership

More information about the members is available in the Global Health Research Directory: researchdirectory.globalhealth.de

**Table:**

Date	January 2020	July 2020	January 2021	July 2021	January 2022	July 2022	January 2023	July 2023	January 2024	July 2024
# Members	92	337	593	688	761	868	969	1060	1115	1263

### B. Running or Completed GLOHRA Projects (as of April 2024)

**Table:**

Project title	Project type	Funder	Research area(s) involved	GLOHRA member institutions^4^	International and/or cross-sector institutions^5^	Partner country
Bladder cancer early detection and treatment in Malawi (EDUROL)	Cross-sector	BMBF (Federal Ministry of Education and Research)	☒Biomedical sciences ☒Public health ☒Social sciences and humanities □Engineering & other sciences	▪ Technische Universität Dresden ▪ University of Münster ▪ Sophien- und Hufeland-Klinikum Weimar	▪ Zomba Central Hospital, Malawi ▪ International Agency for Research on Cancer, France ▪ Rural Surgery Innovations Pvt. Ltd., India	Malawi
Bridging the gap, upgrading the technical know-hows and leveraging research outputs and its dissemination within the Philippines and Germany (Project Buklod)	Partnerships for Research and Training in Health (PATH)	BMZ (Federal Ministry for Economic Cooperation and Development)	N/A	▪ Heidelberg University	▪ Research Institute for Tropical Medicine – Department of Health, Philippines	Philippines
Co-creating one health workforce through health system strengthening in Western India (OHSSIN)	Partnerships for Research and Training in Health (PATH)	BMBF & BMZ	N/A	▪ University of Bonn & University Hospital Bonn	▪ Indian Institute of Public Health Gandhinagar, India ▪ Centre for One Health Education, Research & Development, India	India
Community cervical cancer screening and prevention in Indonesia (IndoCerCa)	Cross-sector	BMBF & BMZ	☒Biomedical sciences ☒Public health □Social sciences and humanities □Engineering & other sciences	▪ Medizinische Hochschule Hannover ▪ University Hospital Münster	▪ Muhammadiyah University of Yogyakarta, Indonesia ▪ Gadjah Mada University Yogyakarta, Indonesia ▪ Dharmais Cancer Hospital, Indonesia	Indonesia
Counselling for alcohol problems in pregnancy (CAP-PRE)	Cross-sector	BMBF	□Biomedical sciences ☒Public health ☒Social sciences and humanities □Engineering & other sciences	▪ Leibniz-Institute for Prevention Research and Epidemiology – BIPS ▪ Technical University of Munich	▪ Federal Centre for Health Education (BZgA), Germany ▪ South African Medical Research Council, South Africa	South Africa
Development of a novel, easy-to-use digital tuberculosis screening tool informed by machine learning approaches (AI TB Screening Tool)	Cross-sector	BMBF & BMZ	☒Biomedical sciences ☒Public health □Social sciences and humanities ☒Engineering & other sciences	▪ Heidelberg University ▪ German Cancer Research Center (DKFZ)	▪ Connected Diagnostics, UK	Kenya Zambia
Diabetes incidence and weight change following dolutegravir transition of ART among PLHIV from an African prospective cohort (INTEGRATIVE)	Global health postdoc fellowship	BMBF	□Biomedical sciences ☒Public health □Social sciences and humanities □Engineering & other sciences	▪ Heidelberg University Hospital	▪ Lighthouse Clinic, Malawi	Malawi
Diagnostic algorithm for peripheral lymph node tuberculosis using portable station (Mobile TB Lab)	Cross-sector	BMBF	☒Biomedical sciences ☒Public health □Social sciences and humanities □Engineering & other sciences	▪ Saarland University Medical Center ▪ Leipzig University ▪ TU Berlin	▪ Midge Medical GmbH, Germany ▪ International Centre for Diarrheal Disease Research, Bangladesh ▪ Makerere University, Uganda	Uganda Bangladesh
Drivers of misinformation, gender gap and masculinity on vaccination decision-making among caregivers in sub-Saharan Africa (MisInfo Vaccines)	Global health postdoc fellowship	BMBF	□Biomedical sciences ☒Public health ☒Social sciences and humanities □Engineering & other sciences	▪ University of Erfurt		Nigeria Malawi Kenya
Education and stewardship to improve knowledge, attitudes and practice towards antimicrobial resistance (ESKAPE AMR)	Partnerships for Research and Training in Health (PATH)	BMBF & BMZ	N/A	▪ Leipzig University & University of Leipzig Medical Center	▪ Kiruddu National Referral Hospital, Uganda	Uganda
Environmental contributions to low colon cancer risk in sub-Saharan Africa: dietary fiber and chronic enteric infections with pathogenic E. coli (DiFiCo)	Global health postdoc fellowship	BMBF	☒Biomedical sciences ☒Public health □Social sciences and humanities □Engineering & other sciences	▪ German Institute of Human Nutrition Potsdam-Rehbruecke (DIfE) ▪ Technical University of Munich	▪ University of Zimbabwe, Zimbabwe ▪ University of Pittsburgh, USA ▪ Stellenbosch University, South Africa	South Africa Zimbabwe Zambia
Human centered design to adapt and inform an integrated chronic disease management program in Uganda using mobile payment services (IMPEDE-CVD)	Cross-sector	BMBF & BMZ	☒Biomedical sciences ☒Public health □Social sciences and humanities ☒Engineering & other sciences	▪ Charité Universitätsmedizin – Berlin ▪ Heidelberg University & Heidelberg University Hospital	▪ mTOMADY GmbH, Germany ▪ ACCESS Uganda, Uganda ▪ Makerere University, Uganda	Uganda
Improving cancer-related health literacy using online stories in sub-Saharan Africa, illustrated by the example of Kenya (CaLioS)	Global health postdoc fellowship	BMBF	☒Biomedical sciences ☒Public health ☒Social sciences and humanities □Engineering & other sciences	▪ University of Freiburg	▪ Kenyatta University, Kenya ▪ Aga Khan University, Kenya ▪ Integrated Cancer Research Foundation of Kenya, Kenya	Kenya
Innovation policies for resilient biomedical R&D systems: lessons from the COVID-19 pandemic innovation response (InnoCOV)	Global health postdoc fellowship	BMBF	□Biomedical sciences ☒Public health ☒Social sciences and humanities □Engineering & other sciences	▪ Heidelberg University Hospital ▪ Bielefeld University ▪ Robert Koch Institute	▪ University of Edinburgh, UK	
Integration of digital mental health intervention at community level in Pakistan (WELLPAK)	Cross-sector	BMBF & BMZ	□Biomedical sciences ☒Public health ☒Social sciences and humanities □Engineering & other sciences	▪ Leibniz-Institute for Prevention Research and Epidemiology – BIPS ▪ Ulm University	▪ Ethno Medizinisches Zentrum e.V., Germany ▪ Fazaia Medical College-Air University, Pakistan	Pakistan
Measuring gender-based discrimination to better understand maternal mortality (MeasureGender)	Interdisciplinary pilot project	BMBF	□Biomedical sciences ☒Public health ☒Social sciences and humanities □Engineering & other sciences	▪ TU Berlin ▪ Heidelberg University Hospital		Burkina Faso Tanzania Ghana
Mosquito-based Artemisinin Resistance Surveillance (MARS)	Global health postdoc fellowship	BMBF	☒Biomedical sciences □Public health ☒Social sciences and humanities □Engineering & other sciences	▪ Charité - Universitätsmedizin Berlin	▪ Université Catholique de Bukavu, DR Congo	Democratic Republic of the Congo
Protecting migrant healthcare workers: closing a gap in Germany’s pandemic preparedness and global health policy (PROTECT)	Interdisciplinary pilot project	BMBF	☒Biomedical sciences □Public health ☒Social sciences and humanities □Engineering & other sciences	▪ Medizinische Hochschule Hannover ▪ University Medical Center Göttingen	▪ Babes-Bolyai University, Romania	Romania
Public health operations for climate change action – development of a framework to identify priority operations (PHONIC)	Cross-sector	BMBF	☒Biomedical sciences ☒Public health ☒Social sciences and humanities □Engineering & other sciences	▪ Ludwig-Maximilians-Universität München ▪ Helmholtz Munich	▪ KLUG – Deutsche Allianz Klimawandel und Gesundheit e.V. Germany ▪ Planetary Health Eastern African Hub (PHEAH), Kenya	Kenya
Racism and mental health: a qualitative study with humanitarian workers (Racism and Mental Health)	Global health postdoc fellowship	BMBF	□Biomedical sciences ☒Public health ☒Social sciences and humanities □Engineering & other sciences	▪ Charité - Universitätsmedizin Berlin	▪ Centre for Planetary Health Policy (CPHP)	
Reducing schistosomiasis through aquaculture interventions in Madagascar: a pilot study (RESAMP)	Interdisciplinary pilot project	BMBF & BMZ	☒Biomedical sciences ☒Public health □Social sciences and humanities ☒Engineering & other sciences	▪ Bernhard Nocht Institute for Tropical Medicine ▪ HAW-Hamburg	▪ APDRA, Madagascar ▪ University of Fianarantsoa, Madagsacar ▪ Université d’Antananarivo ▪ CIRAD, Madagsacar	Madagascar
Safe water – advances in purification options (SWAPNO)	Cross-sector	BMBF	□Biomedical sciences ☒Public health ☒Social sciences and humanities □Engineering & other sciences	▪ Heidelberg University ▪ Potsdam Institute for Climate Impact Research	▪ AGAPE e.V., Germany	Bangladesh
School-based prevention of teacher and family violence: A pilot cluster-randomised controlled trial in Tanzania (PreVio)	Interdisciplinary pilot project	BMBF	□Biomedical sciences ☒Public health ☒Social sciences and humanities □Engineering & other sciences	▪ Bielefeld University ▪ Technical University of Munich	▪ Muhimbili University of Health and Allied Sciences, Tanzania	Tanzania
The contribution of family dynamics and parental mental health to children’s well-being: A 4-year longitudinal study on the mental health of forcibly displaced children (Children’s well-being)	Global health postdoc fellowship	BMBF	□Biomedical sciences □Public health ☒Social sciences and humanities □Engineering & other sciences	▪ Bielefeld University		Iraqi Kurdistan
The impact of helminth infections on vaccine outcomes in humans: a systematic literature review (HelmSys)	Interdisciplinary pilot project	BMBF	☒Biomedical sciences ☒Public health □Social sciences and humanities □Engineering & other sciences	▪ Technical University of Munich ▪ Tübingen University Hospital		
Violence, trust and vaccine hesitancy (ViVac)	Interdisciplinary pilot project	BMBF	□Biomedical sciences ☒Public health ☒Social sciences and humanities □Engineering & other sciences	▪ Universität Hamburg ▪ University of Konstanz ▪ University of Erfurt		Nigeria

^4^ Universities are written in English unless there is no official English name.

^5^ Collaborations are based on the current information provided by project teams, though some adjustments may occur given that many projects are still in progress.

### C. Project Proposals Received

**Table:**

Call for funding	Total applications received	Number of projects funded	Total funding (approximate)
2020-01	14	3	€ 542,000
2020-02	17	4	€ 720,000
2021-01	16	3	€ 600,000
2022-01	19	4	€ 858,000
2022-02	18	4	€ 778,000
2023-01	33	5	€ 854,000
2024-01	51	N/A	N/A
